# Supplementary material for: Enhancing Entomological Surveillance: Real-Time Monitoring of Mosquito Activity with the VECTRACK System in Rural and Urban Areas
Source: Biology (Basel). 2025 Aug 14;14(8):1047. doi: 10.3390/biology14081047 (PMC12383513; doi:10.3390/biology14081047)
Supplement: Supplementary file 1 [file biology-14-01047-s001.zip › biology-3773911-supplementary.pdf]

Table S1. Total number of mosquitoes captured and identified by manual inspections in three different sites by species and sex: in Algarve (coordinates: 37.10134887, −8. 12356948), where the captures occurred between June 24 to September 17 of 2021; in Palmela (coordinates: 38.583248, −8.689730), where the captures occurred between June 8, 2021, to June 23, 2021; in Madeira (coordinates: 32.651318, −16.908259) where the captures occurred between August 8, 2022, to June 28, 2023.

	**Species**	**Algarve**		**Palmela**		**Madeira**			**Total**
		Female	Male	Female	Male	Female	Male	Unknown	
Target	*Aedes albopictus*	15 (1.4%)	0	0	0	0	0	0	15 (1.4%)
	Aedes aegypti	0	0	0	0	65 (6.2%)	83 (7.9%)	12 (0.1%)	160 (15.2%)
	Aedes caspius	0	0	29 (2.8%)	1 (0.1%)	0	0	0	30 (2.8%)
	Culex pipiens	113 (10.7%)	4 (0.4%)	28 (2.7%)	7 (0.7%)	13 (12.4%)	1 (0.1%)	2 (0.2%)	168 (16.0%)
	Culex theileri	0	0	617 (58.7%)	31 (2.9%)	0	0	0	648 (61.7%)
Non-Target	*Culiseta longiareolata*	8 (0.8%)	2 (0.2%)	5 (0.5%)	7 (0.7%)	0	0	0	22 (2.1%)
	Anopheles maculipennis	1 (0.1%)	0	4 (0.4%)	3 (0.3%)	0	0	0	8 (0.8%)
	**Total**	137 (13.0%)	6 (0.6%)	683 (62.1%)	49 (4.7%)	78 (7.4%)	84 (8.0%)	14 (1.3%)	1051
